# Are the Dysnatremias a Permanent Threat to the Critically Ill Patients?

**DOI:** 10.14740/jocmr2425w

**Published:** 2015-12-28

**Authors:** Anibal Basile-Filho, Mayra Goncalves Menegueti, Edson Antonio Nicolini, Alessandra Fabiane Lago, Edson Zangiacomi Martinez, Maria Auxiliadora-Martins

**Affiliations:** a Division of Intensive Care, Department of Surgery and Anatomy, Ribeirao Preto Medical School, University of Sao Paulo, SP 14049-900 Ribeirao Preto, Brazil; b Department of Social Medicine (Statistics), Ribeirao Preto Medical School, University of Sao Paulo, SP 14049-900 Ribeirao Preto, Brazil

**Keywords:** APACHE, Hyponatremia, Hypernatremia, Mortality, Intensive care

## Abstract

**Backgroud:**

The dysnatremias (hyponatremia and hypernatremia) are relatively common findings on admission of intensive care unit (ICU) patients and may represent a major risk. The aim of the study was to assess the ability of serum sodium levels and the Acute Physiology and Chronic Health Evaluation II (APACHE II) to predict mortality of surgical critically ill patients.

**Methods:**

One hundred and ninety-five surgical patients (62% males and 38% females; mean age of 51.8 ± 17.3 years) admitted to the ICU in the postoperative phase were retrospectively studied. The patients were divided into survivors (n = 152) and non-survivors (n = 43). APACHE II, and serum sodium levels at admission, 48 h and discharge were analyzed by generation of receiver operating characteristic (ROC) curves.

**Results:**

The mean APACHE II was 16.3 ± 8.3 (13.6 ± 6.1 for survivors and 25.5 ± 8.5 for non-survivors). The area under the ROC curve for APACHE II was 0.841 (0.782 - 0.889) and 0.721 (0.653 - 0.783), 0.754 (0.653 - 0.783) and 0.720 (0.687 - 0.812) for serum sodium level at admission, 48 h and discharge, respectively.

**Conclusion:**

Even though APACHE II scoring system was the most effective index to predict mortality in the surgical critically ill patients, the serum sodium levels on admission may also be used as an independent predictor of outcome.

## Introduction

The body sodium imbalance may be associated with increased mortality of critically ill patients. Evidence suggests that changes in the serum sodium level on admission to the intensive care unit (ICU) may lead to a poor outcome [1, 2]. Indeed, the dysnatremias (hyponatremia and hypernatremia) are relatively common findings on admission of these patients to the ICU [3, 4], and can affect various physiological organ systems [4-7].

The hypernatremia is an electrolyte disorder and when present can also represent an independent risk factor for mortality. The elevation of serum sodium levels due to, generally, lack of free water, leads to an increase in serum osmolarity, which can alter the distribution of water between intra- and extracellular compartments, and cause intracellular dehydration. These changes can result in adverse effects and contribute to increasing morbidity and mortality [1-6, 8-12]. The hypernatremia acquired during the ICU stay can also act as an independent risk factor for mortality in patients in critical condition [4].

The aim of this study was to assess the ability of serum sodium levels and the Acute Physiology and Chronic Health Evaluation II (APACHE II) to predict mortality of surgical critically ill patients.

## Methods

This study was conducted in a nine-bed adult ICU of Clinics Hospital of Ribeirao Preto Medical School of the University of Sao Paulo (FMRP-USP). This tertiary ICU admits case-mix patients, such as clinical cases in critical condition, transplants and surgical patients in early postoperative phase. The research protocol was approved by the Research Ethics Committee of Hospital das Clinicas da Faculdade de Medicina de Ribeirao Preto (Protocol 7076/2010). Surgical adult patients admitted to the ICU between 2013 and 2014 were analyzed. Data relative to diagnosis upon ICU admission, comorbidities, APACHE II and death risk scores were recorded. Demographic data of groups of patients designated survivors and non-survivors are also reported. Data for calculation of the APACHE II [13] death risk score were collected during the first 24 h after patient admission. Thus, the values of serum sodium measured within 24 h after ICU admission, 48 h after admission and at discharge were collected. Normal serum sodium was defined a serum sodium level between 136 and 144 mmol/L [1, 12, 14, 15].

### Statistical analysis

Comparison of the demographic and clinical data of the patients (survivors versus non-survivors) was accomplished using a Student’s *t*-test for mean value. Variables of demographic and clinical data of the patients (survivors and non-survivors) were expressed as mean ± standard deviation. The capability of each index (APACHE II, sodium admission, sodium 48 h and sodium discharge) to predict mortality of surgical patients was described by receiver operating characteristic (ROC) curves. The area under the ROC curve (AUC) was used as a measure of overall index accuracy, and its significance was tested using the Wilcoxon test. Comparison of the AUC from the different indexes was done using the non-parametric test proposed by DeLong et al [16]. The significance level was set at P < 0.05. Statistical analyses were performed using the MedCalc software version 12.

## Results

This retrospective study included 195 patients (62% males and 38% females; mean age of 51.8 ± 17.3 years) admitted to the ICU in the postoperative phase. The patients were divided into survivors (n = 152) and non-survivors (n = 43). The mean APACHE II was 16.3 ± 8.3 (13.6 ± 6.1 for survivors and 25.5 ± 8.5 for non-survivors, P < 0.05). The death risk (calculated from APACHE II) was 25.3 ± 24.1 (18 ± 17.3 for survivors and 25.5 ± 8.5 for non-suvivors, P < 0.05). Non-oncologic surgery corresponded to 68.7% of admissions to the ICU. The ICU overall mortality was 22%, whereas hospital mortality was 31.7%. When patients were divided into survivors (n = 152) and non-survivors (n = 43), the observed length of stay in the ICU was 3.24 ± 3.8 and 8.2 ± 8.7 days for survivors and non-survivors, respectively (P < 0.05). The use of mechanical ventilation was higher in the non-survivors group when compared with survivors (1.6 ± 3.6 vs. 6.13 ± 6.0 days). Demographic, clinical, and comparison data between survivors and non-survivors are summarized in Table 1.

**Table 1:** General Characteristics of the Surgical Patients Admitted to ICU*

	Patients (n = 195)	Survivors (n = 152)	Non-suvivors (n = 43)
Sex (M/F)	121/74	95/57	26/17
Age (years)	51.8 ± 17.1	51.5 ± 17.3	52.9 ± 16.8
APACHE II score	16.3 ± 8.3	13.6 ± 6.1	25.5 ± 8.5
Death risk (%)	25.3 ± 24.1	18 ± 17.3	50.6 ± 27.7
Mechanical ventilation (days)	2.6 ± 4.8	1.6 ± 3.6	6.13 ± 6.0
ICU length of stay (days)	4.3 ± 5.6	3.24 ± 3.8	8.2 ± 8.7
Hospital length of stay (days)	24 ± 21	23 ± 22	24 ± 20
ICU mortality (%)	22		
Hospital mortality (%)	31.7		
Oncologic surgery	61 (31.3%)	53 (27.2%)	8 (4.1%)
Non-oncologic surgery	134 (68.7%)	99 (50.7%)	35 (18%)
Cardiovascular	18 (9.3%)	13 (6.7%)	5 (2.5%)
Gastrointestinal	114 (58.5%)	86 (44.1%)	28 (14.4%)
Neurosurgery	6 (3%)	5 (2.5%)	1 (0.5%)
Orthopaedic	11 (5.6%)	11 (5.6%)	0
Thorax	19 (9.7%)	14 (7.2%)	5 (2.5%)
Obstetrics and gynecology	9 (4.6%)	9 (4.6%)	0
Urology	17 (8.7%)	13 (6.7%)	4 (2%)
Others	1 (0.5%)	1 (0.5%)	0

*Values expressed as mean ± standard deviation.

**Figure 1:**
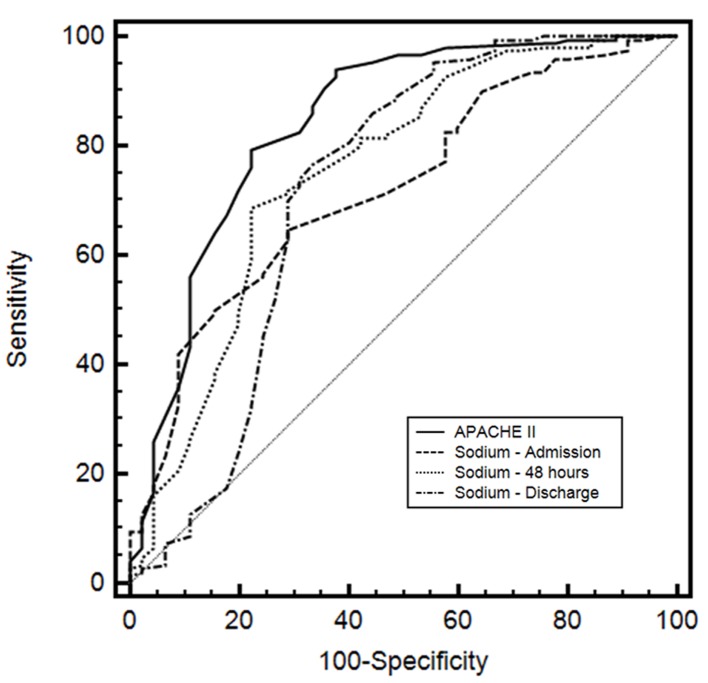
Receiver operating characteristic (ROC) curves of Acute Physiology and Chronic Health Evaluation II (APACHE II), serum sodium level on admission, sodium 48 h after admission and sodium at discharge of patient from ICU.

The AUC for APACHE II was 0.841 (95% CI: 0.782 - 0.889; sensitivity: 79.3; specificity: 77.8). For sodium admission, the AUC was 0.721 (95% CI: 0.653 - 0.783; sensitivity: 64.7; specificity: 71.1). The sodium 48 h and sodium discharge showed an AUC of 0.754 (95% CI: 0.687 - 0.812; sensitivity: 68.7; specificity: 77.8) and 0.720 (95% CI: 0.651 - 0.782; sensitivity: 76.7; specificity: 66.7), respectively. The comparisons of ROC curves for these indexes are depicted in Figure 1 and described in Table 2.

**Table 2:** Comparison of Areas Under the Curve (AUC) of Receiver Operating Characteristic (ROC) for Different Predictors at ICU

Variable	AUC	95% CI	P value
APACHE II	0.841	0.782 - 0.889	0.03
Sodium admission (mmol/L)	0.721	0.653 - 0.783	0.04
Sodium 48 h (mmol/L)	0.754	0.687 - 0.812	0.04
Sodium discharge (mmol/L)	0.720	0.651 - 0.782	0.05

Hypothesis test of diagnostic accuracy P < 0.05 (Wilcoxon test) [15].

The hyponatremia (Na < 136 mmol/L), normal sodium levels (136 - 144 mmol/L) and hypernatremia (Na > 144 mmol/L) represented 34.9% (64/4, survivors and non-survivors), 43.4% (78/26) and 11.7% (10/13) of patients, respectively (Table 3).

**Table 3:** Distribution of Different Serum Sodium Levels Between Survivors and Non-Survivors Present on Admission of the Surgical Patient to ICU

Patients (n = 195)	Na < 136*	Na 136 - 144	Na > 144
Sex (M/F)	44/24	62/42	14/9
Age (years)	55 ± 13	50 ± 14	50 ± 13
APACHE II score	14 ± 5	21 ± 14	22 ± 6
Survivors	64	78	10
Non-survivors	4	26	13
n	68	104	23
Percentage (%)	34.8	53.4	11.7

*Values expressed as mmol/L.

**Figure 2:**
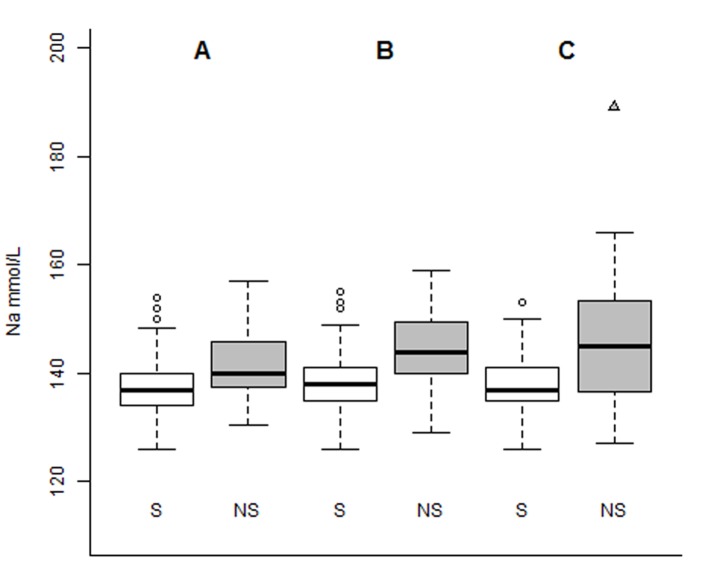
Boxplot of serial serum sodium levels (in mmol/L), distributed by groups of patients of survivors (S) and non-survivors (NS) for sodium admission (A), sodium 48 h (B) and sodium discharge (C).

As demonstrated below in Figure 2, the median values for sodium admission, sodium 48 h and sodium discharge were 137, 138 and 137 mmol/L for survivors and 140, 144 and 145 for non-survivors, respectively.

## Discussion

This restrospective study, based on a binary outcome (survivors and non-survivors) modeling, was designed to investigate the ability of sodium serum levels and APACHE II to predict mortality of surgical critically ill patient.

Hyponatremia is usually defined as a value of serum sodium < 130 mmol/L [17]. This electrolyte imbalance is undoubtedly the most common in clinical practice in all medical services. DeVita et al [8] have demonstrated an incidence of hyponatremia in the ICU of about 30%, whereas Bennani et al [18] in a cohort of 2,188 patients found that 300 of them (13.7%) with hyponatremia on admission. However, other studies showed a higher incidence of hyponatremia of 34.3% [19]. These data are comparable to those demonstrated in our study showing a high incidence of hyponatremia on admission to ICU (34.9%). Hyponatremia can occur due to organic dysfunction (liver or heart failure), adrenal insufficiency, diuretic use and inappropriate secretion of the antidiuretic hormone (ADH) [5]. Additionally, hyponatremia is a predictor of increased mortality in several diseases such as congestive heart failure, community-acquired pneumonia, and even in case-mix hospitalized patients [20]. Moreover, nausea, pain, stress, and infusion of volume have a tendency to increase levels of ADH in the preoperative phase and may enhance the incidence of hyponatremia [21]. Darmon et al [2] demonstrated that mild hyponatremias (serum sodium value ≥ 125 and < 130) are independently associated with worse prognosis of patients, adjusted for severity and comorbidities. These authors pointed out the prognostic consequences of borderline dysnatremias. Padhi et al [19] found in their study that patients with hyponatremia had a longer ICU length of stay (P = 0.02), and days of mechanical ventilation (P < 0.05) with consequent increased rate of mortality (P = 0.01), compared to patients with normal serum sodium. Leung et al [22] studied a total of 75,423 patients with preoperative hyponatremia (serum sodium < 135 mEq/L) and compared with 888,840 patients with sodium levels within the normal range (135 - 144 mEq/L). These authors stated that preoperative hyponatremia was associated with a higher risk of 30 days postoperative mortality (5.2% versus 1.3%; odds ratio (OR): 1.44; 95% CI: 1.38 - 1.50).

The hypernatremia is defined to a serum sodium level > 144 mEq/L [15]. Since the serum sodium is determined by the ratio between the amount of sodium in the serum and the amount of water in the plasma, hypernatremia appears from any excess of sodium such as the administration of hypertonic fluids, hypotonic fluid loss (water free) or a combination of both. Several factors can predispose the ICU patient to hypernatremia as lack of free water, the administration of hipertonic solutions, renal water loss, the use of diuretics, fluid losses through gastrointestinal drainage, fever, fistulas and open wounds [23]. Although less frequent than hyponatremia, some studies have shown an incidence of hypernatremia of 2.5% in ICU admissions [24, 25]. In the present study, 12% of patients presented hypernatremia on intensive care admission and the mortality was very high (56.5%, i.e., 13 out of 23 patients). Mortality rates in hypernatremia patients, especially those in ICU, are very high, ranging from 15% to 50%, depending on the severity of this disorder [26, 27]. Lindner et al [4] evaluated 981 critical patients and identified in a multivariate analysis that hypernatremia played a role of an independent risk factor for mortality and relative risk of 2.1 (95% CI: 1.4 - 3.3). Bihari et al [14] while investigating 436,209 patients admitted to ICU found that patients with hypernatremia (serum sodium ≥ 160 mmol/L) had a higher chance of death, with OR of 4.2 and 95% CI of 3.6 - 4.9. However, these authors did not find differences in the chances of death in patients with normal sodium compared with hypernatremia in patients with respiratory diseases. Additionally, Waite et al [28] demonstrated in 207,702 critical patients, an incidence of 4.3% of hypernatremia and its relationship as independent risk to predict mortality with a relative risk of 1.34 and 95% CI of 1.4 - 1.45. The authors have also shown that the risk of death increases for severe hypernatremia. However, the duration of hypernatremia did not influence mortality.

The comparison of serum sodium and APACHE II as a predictor of mortality, the AUC for the APACHE II, sodium admission and sodium 48 h was 0.841, 0.721 and 0.754, respectively. These results demonstrate that APACHE II in this study performed better in the prediction of mortality. Even though APACHE II is one of the most common prognostic index used in the ICU worldwide, this score system employs multiple variables such as 12 routine physiologic measurements, age, comorbidities, elective or postemergency surgery, Glasgow coma scale and diagnostic category weight [13]. Therefore, serum sodium levels migth be used as a single predictor of mortality in surgical patients.

The present cohort of 195 patients was able to detect that persistence of hypernatremia, even borderline (Fig. 2), showed a very high mortality (10/23 patients: 56.5%, Table 3) and therefore can play an independent role in mortality prediction of critically ill patients. However, further studies with a larger cohort should be carried out to detect the impact of dysnatremias on survival rates of surgical criticallly ill patients.

### Conclusion

The results of the present investigation demonstrated the prognostic ability of the APACHE II scoring system for prediction of the mortality of surgical critically ill patients upon ICU admission. Additionally, even though APACHE II was the most effective index to predict mortality, and hypernatremia on admission may also be used as an independent risk factor. Furthermore, these data suggest that the goal to increase the chances of survival of patients is to adjust serum sodium levels, especially hypernatremia, that have a relationship with mortality of these patients.
